# How to Secure Safe and Successful Vaccination

**Published:** 1900-03

**Authors:** 


### How to Secure Safe and Successful Vaccination.

A number of methods have been used in vaccinating—the following being one of the simplest and most successful:

Cleanse the site of inoculation with *soap and water,* and carefully dry.

(Do not use bichloride solution, carbolic acid, or any other disinfectant, unless there are special reasons for doing so—in which case all traces of such disinfectant should be removed with sterilized water before vaccinating.)

Take the small rubber in the left hand, with the neck of the bulb pointing to the left. One of the sealed glass tubes held in the right hand is inserted into the opening in the top or rounded end of the bulb, and, with a slight rotary motion, pushed through until the bulb is impaled and resting at about the center of the tube. The end of the tube to the right is then broken off and the tube pushed on through until the open end is well within the cavity of the bulb. Now break off the other end of the tube, and lay the tube aside until the site is scarified.

Scarify a single small area—say about one-quarter or one-third of an inch in diameter—using for this purpose almost any sterile steel instrument, such as a needle, scalpel, or one of the many instruments especially designed for this work.

To apply vaccine, hold the bulb between the thumb and second finger, the tip of the index finger covering the air-hole at the top, when slight pressure upon the bulb will force the vaccine out upon the scarified area. The vaccine should then be *rubbed in thoroughly* with the flat side of the instrument, with an *occasional slight scratching or pricking with the point,* in order to facilitate *penetration* and absorption.

Ample time should be given for the lymph to dry, and the clothing should never be replaced until the site presents a glazed or varnished appearance. No dressing is required except in cases where very rough or dirty under-garments are worn, and then a piece of clean, soft linen or cotton is the most suitable.

### A FEW DON'TS FOR THE OPERATOR.

1. *Don't prepare the site by washing with antiseptic solutions.* Or if this should be necessary—

2. Don't fail to rinse thoroughly with sterilized (boiled) water, and dry.

3. *Don't draw blood if you can help it. A gentle oozing of serum gives much better results.*

4. Don't fail to *rub the vaccine thoroughly and persistently into the abrasion.*

5. Don't replace the clothing *until the vaccine is thoroughly dry.*

6. Don't apply antiseptic dressings. (Many of the most successful vaccinators never use any dressing except in cases where there is danger of infection from the environment or uncleanliness of the patient.)

7. Don't expose vaccine to extremes of temperature. High temperatures spoil it.

8. Don't expect to find a swollen arm, indurated glands, high fever and a suppurating ulcer—these belong to the old-fashioned means and methods of vaccinating.

9. Don't accept the word of the patient or parent as to the success or failure of the process. Examine the case yourself, and if you find a typical vesicle—or the remains of one or more that may have been ruptured and emptied—assure the patient that he is protected against smallpox.

10. Don't be in a hurry about passing judgment upon a "take." Sometimes the vesicles are delayed in their development.

